# Baseline Food Supply-Based Dietary Scores and Ischemic Heart Disease Mortality: A Longitudinal Cross-Country Study

**DOI:** 10.3390/nu18203456

**Published:** 2026-10-21

**Authors:** Saya Nosaka, Fumiya Kawase, Tomoko Imai, Ayako Sezaki, Keiko Miyamoto, Chisato Abe, Masayo Sanada, Norie Sugihara, Toshie Honda, Yuta Sumikama, Ayaka Inden, Takayoshi Tsukahara, Hiroshi Shimokata

**Affiliations:** 1 Graduate School of Nutritional Science, Nagoya University of Arts and Sciences, Aichi 470-0196, Japan; saya.nosaka@gmail.com (S.N.); 20gn001@st.nuas.ac.jp (Y.S.); ttsuka@nuas.ac.jp (T.T.); 2 Department of Nutrition, Osaka Metropolitan University, Osaka 536-8525, Japan; 3 Department of Sports and Health Sciences, Graduate School of Biomedical Engineering, Tohoku University, Sendai 980-8575, Japan; 4 Institute of Health and Nutrition, Nagoya University of Arts and Sciences, Nisshin 470-0196, Japan; toimai@dwc.doshisha.ac.jp (T.I.); ms06lantha@gmail.com (A.S.); hike5843@yahoo.co.jp (K.M.); masasana311@gmail.com (M.S.); honda@nuas.ac.jp (T.H.); ayaka15@hama-med.ac.jp (A.I.); simokata@nuas.ac.jp (H.S.); 5 Department of Food Science and Nutrition, Doshisha Women’s College of Liberal Arts, Kyoto 602-0893, Japan; 6 Center for Nutritional Epidemiology and Policy Research, National Institutes of Biomedical Innovation, Health and Nutrition, Settsu 566-0002, Japan; 7 Department of Nursing, Nagoya University of Arts and Sciences, Nagoya 460-0001, Japan; 8 Department of Food and Nutrition, Tsu City College, Tsu 514-0112, Japan; 9 Department of Nursing, Heisei College of Health Sciences, Gifu 501-1131, Japan; 10 Faculty of Health and Social Services, Kanagawa University of Human Services, Yokosuka 238-8522, Japan; notch7.8@gmail.com; 11 Department of Nutrition, Japanese Red Cross Aichi Medical Center Nagoya Daiichi Hospital, Nagoya 453-8511, Japan; 12 Clinical Nutrition Unit, Hamamatsu University Hospital, Hamamatsu 431-3192, Japan; 13 Department of Nutritional Science, Nagoya University of Arts and Sciences, Nisshin 470-0196, Japan

**Keywords:** ischemic heart disease, dietary patterns, diet quality, ecological study, FAOSTAT

## Abstract

**Background/Objectives:** National food environments may be associated with cardiovascular risk, but how their association with country-level ischemic heart disease (IHD) mortality differs across diet-quality indices remains unclear. We compared six food supply-based dietary scores calculated using 2010 food data in relation to annual IHD mortality from 2010 to 2021. **Methods:** We conducted a longitudinal study of 148 countries with populations ≥ 1 million. Using 2010 FAOSTAT Food Balance Sheet data, we calculated the Dietary Approaches to Stop Hypertension (DASH) score, the Alternative Healthy Eating Index (AHEI), Mediterranean Diet Score (MDS), Traditional Japanese Diet Score (TJDS), Planetary Health Diet Index (PHDI), and Quantitative Index for Dietary Diversity (QUANTIDD). The outcome was age-standardized IHD mortality during 2010–2021. Associations between time-invariant baseline dietary scores and annual IHD mortality were assessed using multivariable-adjusted linear mixed-effects models. Effect sizes were expressed as mortality rate ratios (MRRs) per 1-standard deviation (SD) higher score; model fit was compared using Akaike information criterion (AIC) weights and marginal R^2^, and effect estimates were compared pairwise. **Results:** Higher DASH and AHEI scores were associated with lower IHD mortality. MRRs per 1-SD higher score were 0.914 (95% confidence interval, 0.849–0.983) for DASH and 0.908 (95% confidence interval, 0.838–0.985) for AHEI. No significant associations were observed for MDS, TJDS, PHDI, or QUANTIDD. DASH had the highest AIC weight (0.434), followed by AHEI (0.341), with a slightly higher marginal R^2^ for DASH. Pairwise comparisons showed no statistically significant differences in effect estimates between DASH and AHEI or between either of these scores and the other dietary scores. **Conclusions:** Higher baseline DASH and AHEI scores were associated with lower IHD mortality during 2010–2021, with similar effect estimates per 1-SD higher score and broadly comparable model fit. However, direct pairwise comparisons revealed no statistically significant differences in standardized effect sizes between these scores (DASH and AHEI) and the other dietary scores. These findings suggest that the relevance of dietary scores may depend on the outcome and analytic context rather than the superiority of a single index. Because this was an ecological study, these findings should not be interpreted as individual-level causal effects.

## 1. Introduction

Ischemic heart disease (IHD) remains the leading cause of death worldwide [1] and continues to impose a substantial burden on healthcare systems despite major advances in pharmacological therapy and surgical interventions [2]. IHD mortality varies substantially across countries and regions, potentially reflecting multiple population-level factors, including healthcare systems, socioeconomic conditions, lifestyles, and food supply structures [3]. An unhealthy diet is a major modifiable risk factor for cardiovascular disease, and improving diet is an important strategy for cardiovascular disease prevention at the individual and population levels [4,5]. However, diet quality does not depend solely on an individual’s preference but is related to national food systems and environments, including food supply and availability, prices, distribution, retail, and processing [6,7]. Food environments worldwide are diverse, with increased supplies of ultra-processed and energy-dense foods coexisting with undernutrition and micronutrient deficiencies. Food supply structures that can support healthy diets differ substantially across countries and regions. Therefore, to understand the international differences in IHD mortality, it is important to examine not only individual-level dietary intake but also national food supply structures that may support healthy eating from public health and nutrition policy perspectives.

Representative dietary scores used to quantify diet quality include the Dietary Approaches to Stop Hypertension (DASH) score, Alternative Healthy Eating Index (AHEI), Mediterranean Diet Score (MDS), Traditional Japanese Diet Score (TJDS), Planetary Health Diet Index (PHDI), and Quantitative Index for Dietary Diversity (QUANTIDD). However, these dietary scores differ in their development objectives, components, and underlying food cultures, and their associations with health outcomes may differ even within the same population [8,9,10].

Comparative studies applying multiple dietary scores to the same data have primarily been conducted as individual-level cohort studies or international comparative studies describing global distributions of dietary intake and changes over time [9,11,12,13]. In contrast, country-level ecological studies using FAOSTAT data have examined associations of Japanese dietary scores and MDSs with IHD and mortality [10,14,15], and more recently, multiple supply-based dietary diversity indices have been compared longitudinally [16]. However, to our knowledge, few studies have calculated multiple dietary scores using national food supply data and compared their associations with IHD mortality using standardized effect estimates and model-fit statistics across economically and geographically diverse countries within a common analytical framework.

The FAOSTAT Food Balance Sheets provide standardized long-term time-series data on average national food supply conditions and are widely used for international comparisons and assessments of temporal trends. However, they do not adequately reflect waste at the consumption stage, cooking losses, or the distribution of intake among individuals and therefore do not represent actual individual dietary intake. Despite these limitations, they are useful for ecological studies examining associations between national food supply structures and health indicators [10,14,15,17,18,19].

Accordingly, in this study, internationally comparable FAOSTAT food supply data were used to characterize national food supply structures in 2010, and six supply-based dietary scores were calculated as baseline exposures. Under a common analytical framework, we aimed to compare the associations of these baseline dietary scores with annual IHD mortality during 2010–2021 using standardized effect estimates and model-fit statistics. The study evaluated whether cross-country differences in national food supply patterns at baseline were associated with IHD mortality over the study period, rather than whether temporal changes in diet quality were associated with changes in mortality. As a supplementary analysis, we evaluated the associations between each dietary score and all-cause mortality to determine whether the patterns observed for IHD mortality differed from those for overall mortality.

## 2. Materials and Methods

### 2.1. Study Design

This cross-country study used publicly available country-level data, with baseline dietary scores calculated from 2010 food supply data and mortality assessed annually from 2010 to 2021. We included 148 countries with populations of at least 1 million for which complete data were available for baseline dietary scores and covariates in 2010 and for country-specific age-standardized IHD mortality from 2010 to 2021. The dietary scores and covariates were treated as time-invariant baseline variables, whereas mortality was assessed annually. All data were country-level aggregate data, with country-year observations used as the unit of analysis for the repeated mortality models. The study used only publicly available aggregate data and did not include personally identifiable information.

### 2.2. Outcomes

The primary outcome was the country-specific age-standardized IHD mortality rate per 100,000 population from the Global Burden of Disease Study (GBD) 2021. As a supplementary outcome, all-cause mortality during the same study period was examined [20]. The GBD is a large-scale scientific collaboration led by the Institute for Health Metrics and Evaluation at the University of Washington, USA, that aims to quantify and compare the burden of diseases, injuries, and risk factors by age, sex, and location.

### 2.3. Dietary Data and Scoring Methods

Food supply data for each country were obtained from FAOSTAT as mean per capita food supply (g/day) and mean energy supply (1000 kcal/day) [21]. Sodium intake data used to calculate the selected dietary scores were obtained from the GBD 2023 covariates [22]. FAOSTAT provides food and agricultural data for countries and regional groups worldwide. In the FAOSTAT Food Balance Sheets, food supply is estimated as the amount available for human consumption at the retail level after deducting losses occurring along the supply chain from the post-harvest or post-slaughter stages through retail. However, these estimates do not directly represent actual individual intake because they do not fully account for waste and losses occurring at the retail and consumer stages [23]. The year 2010 was used as the baseline. After duplicate food items were removed, food items were classified into the component food groups of each dietary score, with sodium incorporated separately using GBD 2023 covariate data when required by the definition of the respective dietary score [22].

### 2.4. Diet Scores

Six dietary indices were used: DASH, AHEI, MDS, TJDS, PHDI, and QUANTIDD. Brief descriptions of each index are provided below. Their scoring methods are summarized in Supplementary Table S1, and detailed component definitions and scoring criteria are provided in Supplementary Tables S2–S7.

#### 2.4.1. DASH

The DASH diet is a dietary pattern developed to lower blood pressure, and its antihypertensive effects have been demonstrated in the original DASH trial and the DASH-Sodium trial, which combined the DASH diet with sodium reduction [24,25]. Subsequently, dietary scores were developed to evaluate adherence to the DASH dietary pattern, and their associations with health outcomes, including coronary heart disease, were examined [26]. In this study, the DASH score was calculated based on the index developed by Miller et al. to assess adherence to the DASH dietary pattern [11]. Foods included in FAOSTAT were classified into eight DASH food groups, and the score was calculated based on food supply per 1000 kcal in each country. For the five components for which a higher supply was desirable (fruits, vegetables, total grains, nuts and legumes, and milk and dairy products), countries were classified into quintiles from the lowest to highest supply and assigned 1–5 points. For the three components for which a lower supply was desirable (sodium, meat, and sugar), countries were classified into quintiles from the highest to lowest supply and assigned 1–5 points. However, because the data used in this study did not allow us to determine the supply of whole grains or low-fat milk as specified in the original index, these components were replaced with total grains, without distinguishing whole grains from refined grains, and milk and dairy products, without distinguishing low-fat from regular milk. Similarly, sugar-sweetened beverages were replaced with total sugar. Moreover, because FAOSTAT food supply data cannot be disaggregated by sex, the DASH score calculated in this study was a common country-level value for both sexes. The total DASH score was calculated by summing the scores for the eight components and ranged from 8 to 40 points. A higher total score indicates a healthier dietary pattern and greater adherence to the DASH diet.

#### 2.4.2. AHEI

The AHEI evaluates foods and nutrients associated with chronic disease risk using absolute criteria [9,27,28]. In this study, adherence to the AHEI dietary pattern was calculated based on the international scoring method used by Miller et al. [11]. For each component, scores were assigned according to the supply level based on prespecified recommended supply criteria, with 0 points assigned to the least desirable supply level and 10 points to the most desirable supply level. The relative scoring criteria for the sodium component were based on the 10th and 90th percentiles of the energy-adjusted sodium supply distribution in 2010. However, because whole grains and refined grains cannot be distinguished in FAOSTAT food supply data, the whole-grain component used in the original index was replaced with total grain supply, without distinguishing whole grains from refined grains. In addition, although the original index specifies sex-specific cutoffs for whole grains, FAOSTAT food supply data cannot be disaggregated by sex; therefore, we used the mean of the male and female cutoff values. Furthermore, because the supply of sugar-sweetened beverages and fruit juices, as defined in the original index, could not be determined directly, the supply of sugars and sweeteners was used as a proxy. Similarly, fish oil supply was used as a proxy for the seafood omega-3 fatty acid component and was converted from g/capita/day to mg/capita/day, whereas selected vegetable oil supply was used as a proxy for the polyunsaturated fatty acid component and expressed as a percentage of total energy supply, assuming 9 kcal/g. Component scores were summed to calculate the AHEI score, which ranged from 0 to 90. A higher total score indicates a healthier dietary pattern and greater adherence to the AHEI.

#### 2.4.3. MDS

The Mediterranean diet refers to the traditional dietary habits of countries bordering the Mediterranean Sea. To assess adherence to the Mediterranean diet, we used a partially modified version of the MDS proposed by Trichopoulou et al. [15,29], following the approach used in a previous study by our research group [15]. FAOSTAT food supply data were classified into eight food groups: six considered beneficial to health (vegetables, legumes, fruits and nuts, grains, fish, and olive oil) and two for which a lower supply was considered desirable (meat and dairy products). Scores were calculated based on food supply per 1000 kcal for each country. For the six food groups considered beneficial to health, 1 point was assigned when the supply was at or above the median of the study population and 0 points when the supply was below the median. The food groups for which a lower supply was considered desirable were reverse-scored. As in a previous study, olive oil supply was used instead of the ratio of monounsaturated to saturated fatty acids used in the original MDS. In the present study, alcohol (wine), which was included in the original score, was excluded because recent evidence has questioned its cardiovascular benefits and indicated potential cardiovascular risks [30,31,32]. In addition, only a limited number of countries met the conventional criterion for moderate supply. Component scores were summed to calculate the MDS, which ranged from 0 to 8. A higher total score indicates greater adherence to the Mediterranean diet.

#### 2.4.4. TJDS

The Japanese diet refers to traditional Japanese dietary habits in which rice is the staple food. To evaluate adherence to a traditional Japanese dietary pattern, we used the revised eight-item version of the TJDS reported in previous studies [10,14]. Based on the scoring approach of the MDS proposed by Trichopoulou et al. [29], the TJDS comprises eight food groups that reflect the characteristics of the traditional Japanese diet. The supply of each food group in each country was calculated per 1000 kcal and classified into tertiles according to supply level. For the five food groups commonly consumed in the traditional Japanese diet (rice, fish, soybeans, vegetables, and eggs), +1, 0, and −1 points were assigned from the highest to the lowest supply group. For the three food groups consumed relatively less in the traditional Japanese diet (wheat, milk, and red meat), −1, 0, and +1 points were assigned from the highest to the lowest supply group. The total TJDS was calculated by summing the scores for the eight food groups and ranged from −8 to 8 points. A higher total score indicates that a country’s dietary pattern more closely resembles the traditional Japanese dietary pattern.

#### 2.4.5. PHDI

The Planetary Health Diet is a dietary pattern proposed by the EAT–Lancet Commission to jointly promote human health and environmental sustainability [33]. In this study, to evaluate adherence to the Planetary Health Diet, we referred to the PHDI developed by Bui et al. [34] and specified criteria corresponding to a score of 0 and the maximum score for each of the 15 components. The eight components for which a high supply is desirable are fruits, vegetables, grains, legumes, nuts, soybeans, fish and seafood, and unsaturated oils. The seven components for which a lower supply is desirable are potatoes, red and processed meat, poultry, eggs, milk and dairy products, sugar, and saturated fat. However, several components required modification because of limitations in the FAOSTAT food supply data. Whole grains were replaced with total grains because whole grains could not be distinguished from refined grains. Added sugars and sugars from fruit juice were represented by total sugar and sweetener supply. Each component was scored proportionally according to the supply between the criterion corresponding to 0 points and the criterion corresponding to the maximum score. The maximum score for each component was generally 10 points; however, the scores for legumes and soybeans were each multiplied by 0.5, yielding a maximum of 5 points for each. The total PHDI was calculated by summing the scores of the 15 components and ranged from 0 to 140 points. A higher total score indicates greater adherence to the EAT–Lancet reference diet and a dietary pattern that considers human health and environmental sustainability.

#### 2.4.6. QUANTIDD

Dietary diversity has been reported to be associated with a variety of health outcomes, including cardiovascular disease, cardiometabolic risk, and healthy life expectancy [35,36,37]. In this study, we used QUANTIDD, proposed by Katanoda et al., as a reference measure to quantitatively assess dietary diversity [38]. Following the approach used in our previous study [37], food group proportions were calculated on a weight basis using food supply quantity rather than on an energy basis, as in the original QUANTIDD. Foods were classified into 12 groups: potatoes, vegetables, legumes, nuts and seeds, fruits, dairy products, grains, meat, fish, eggs, oils and fats, and sugars and sweeteners. Let *pi* denote the proportion of the total supply contributed by each food group, and *n* denote the number of food groups. 

QUANTIDD was calculated using the following equation:

$$\mathrm{QUANTIDD}=\frac{1-\sum_{i=1}^{n}p_{i}^{2}}{1-\frac{1}{n}}$$

QUANTIDD evaluates the evenness of the distribution of food supply across food groups based on the proportion of total supply contributed by each food group. Scores range from 0 to 1; lower values indicate that food supply is concentrated in particular food groups, whereas higher values indicate a more even distribution across food groups and greater diversity in the food supply.

### 2.5. Covariates

To account for potential confounding factors, we included baseline country-level socioeconomic, demographic, lifestyle, and environmental factors associated with IHD mortality. Gross domestic product (GDP; US$1000 per capita) and the proportion of the population aged ≥ 65 years (%) were obtained from the World Bank database [39]. Total energy supply (1000 kcal/person/day) was obtained from FAOSTAT [21]. Years of education (years), smoking prevalence (%), physical activity (1000 MET-min/week), PM2.5 (µg/m^3^), obesity prevalence (body mass index ≥ 30 kg/m^2^; %), and latitude were obtained from GBD 2021 [20].

### 2.6. Statistical Analysis

For the baseline data in 2010, the distribution of each variable was described using the mean, standard deviation (SD), and 5th, 25th, 50th, 75th, and 95th percentiles. Country-specific dietary scores for 2010, which were used as exposure variables in the primary analysis, are presented in Supplementary Table S8.

For the analysis of repeated annual mortality data from 2010 to 2021, the outcome was defined as the natural logarithm of the IHD mortality rate. Covariate-adjusted linear mixed models (LMMs) were used. Dietary scores and baseline country-level covariates were treated as time-invariant variables measured in 2010. Year was included as a continuous time variable, and annual mortality rates from 2010 to 2021 were treated as time-varying outcomes. The unit of analysis was the country-year observation. To account for repeated annual observations within countries, year was included as a fixed effect to account for the overall temporal trend, and each model included a country-specific random intercept and random slope for year. Baseline dietary scores and continuous country-level covariates were mean-centered by subtracting their respective means across the included countries. Year was also mean-centered across the observation period. The outcome variable was not mean-centered. To compare model fit among dietary scores, LMMs were fitted using maximum likelihood, and Akaike information criterion (AIC) weights were calculated from these models. Marginal R^2^ was evaluated as a supplementary indicator of model fit. The lmer function in the lmerTest package was used for the LMM analyses [40].

To compare the magnitudes of the associations among the dietary scores, we calculated the mortality rate ratio (MRR) per 1-SD increase in each dietary score. Because the outcome variable was the natural logarithm of the mortality rate, the 1-SD MRR was calculated as exp (β × SD), where β represents the regression coefficient for each dietary score and SD represents its standard deviation. The 95% confidence intervals (CIs) were calculated using the corresponding standard errors. A forest plot was constructed using the resulting 1-SD MRRs. To assess whether the associations between baseline dietary scores and IHD mortality varied over the years, additional models including a dietary score × year interaction term were fitted using the same covariate structure as in the primary analysis. Because dietary scores were calculated from 2010 food supply data and treated as fixed baseline exposures, these interaction terms were interpreted as temporal variation in the association between baseline dietary scores and annual IHD mortality over the study period, rather than as effects of changes in diet over time. Pairwise differences in the standardized log-MRRs were further evaluated using a country-level cluster bootstrap with 2000 resamples, in which countries were resampled with replacement while retaining all annual observations within each sampled country. Nine pairwise comparisons involving DASH and/or AHEI were examined, and *p* values were adjusted for multiple comparisons using the Holm method.

In addition, sensitivity analyses were conducted to assess the robustness of the primary findings. First, to strengthen the temporal separation between baseline dietary exposure and the outcome, the analyses were repeated while excluding IHD mortality observations for 2010 and using annual IHD mortality data from 2011 to 2021. Second, to assess the potential influence of over-adjustment, the analyses were repeated without controlling for obesity prevalence. Third, potential extreme values in baseline dietary scores were assessed using Tukey’s outer-fence criterion, defined as values below Q1 − 3 × interquartile range (IQR) or above Q3 + 3 × IQR. Only one country met this criterion for QUANTIDD; therefore, an additional sensitivity analysis excluding this country was conducted for QUANTIDD. Fourth, the models were additionally controlled for alcohol consumption. Fifth, the models further controlled for the proportion of the Muslim population. As a supplementary analysis, all-cause mortality was analyzed using the same analytical framework as that used for IHD mortality; however, these results were interpreted as supplementary findings.

Statistical significance was defined as *p* < 0.05, and all analyses were performed using R software (version 4.5.3; R Foundation for Statistical Computing, Vienna, Austria) [41].

## 3. Results

### 3.1. Distribution

**Figure 1:**
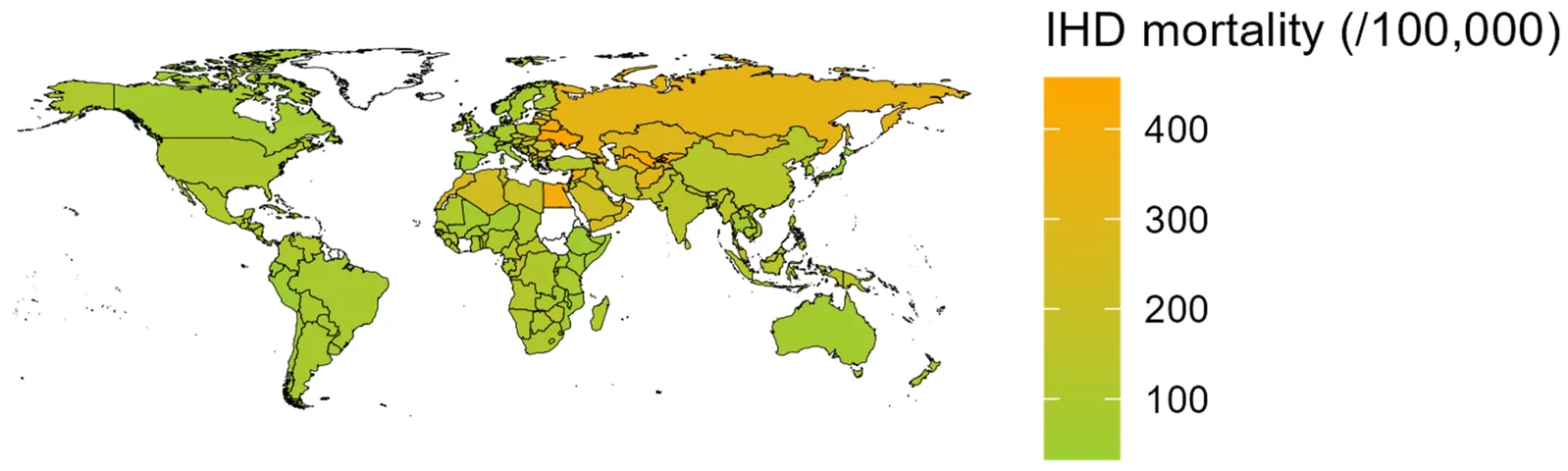
World map of age-standardized ischemic heart disease (IHD) mortality rates in 2010. Lower mortality rates are shown in yellow-green, whereas higher mortality rates are shown in orange.

Table 1 shows the mean, SD, and percentile values in 2010 for the six dietary scores, IHD mortality, confounders, and sodium intake across 148 countries worldwide. Marked geographical differences in IHD mortality rates were observed in 2010. The countries with the highest IHD mortality rates were Ukraine (456.884), Uzbekistan (431.892), and Egypt (409.181). The countries with the lowest IHD mortality rates were Japan (32.634), South Korea (38.011), and Taiwan (38.390) (Supplementary Table S9). On the world map, countries with high IHD mortality rates were concentrated across Eurasia and the Middle East, whereas IHD mortality rates were relatively low in much of East Asia, Western Europe, and North America (Figure 1).

### 3.2. Associations Between Baseline Dietary Scores and IHD Mortality from 2010 to 2021

Associations between baseline dietary scores and IHD mortality were examined using linear mixed-effects models. In the fully adjusted model, higher baseline DASH and AHEI scores were associated with lower IHD mortality rates during the 2010–2021 study period. For DASH, β was −0.024 (standard error [SE] = 0.010, *p* = 0.016), and the 1-SD MRR was 0.914 (95% CI, 0.849–0.983). For AHEI, β was −0.011 (SE = 0.005, *p* = 0.021), and the 1-SD MRR was 0.908 (95% CI, 0.838–0.985). Thus, a 1-SD higher baseline DASH or AHEI score was associated with approximately 10% lower IHD mortality during the study period.

**Figure 2:**
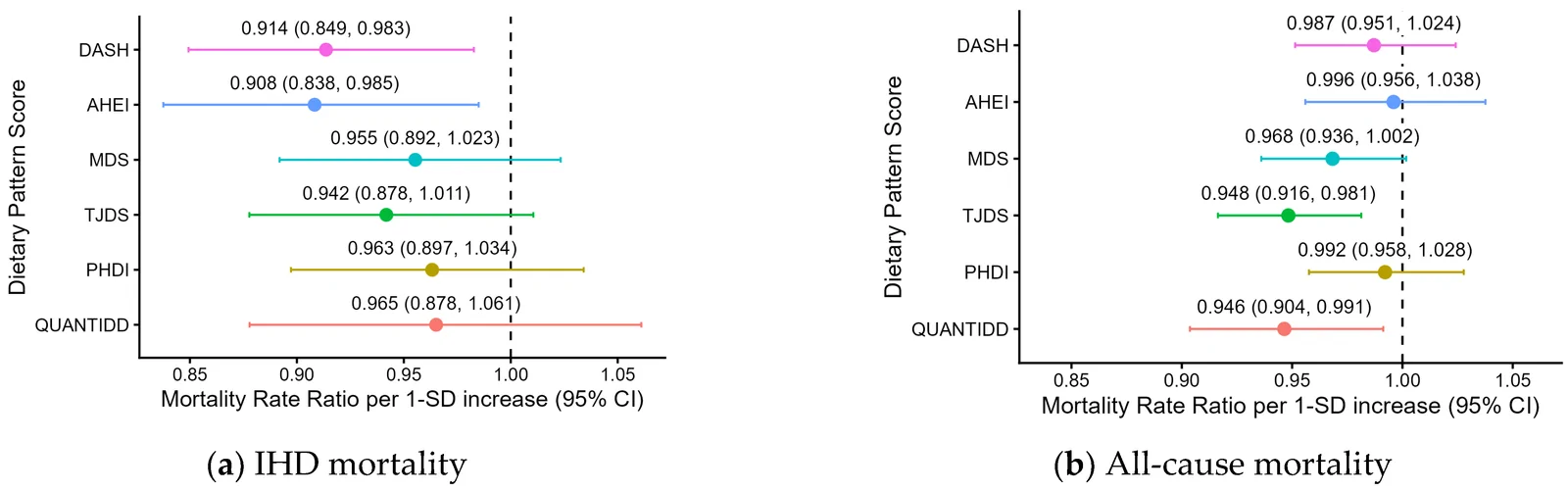
Forest plots of mortality rate ratios and 95% confidence intervals for mortality associated with a 1-SD increase in each dietary score. Mortality rate ratios (MRRs) were estimated for a 1-SD increase in each dietary score. An MRR < 1 indicates a lower mortality rate associated with a higher dietary score. SD, standard deviation; MRR, mortality rate ratio; CI, confidence interval; IHD, ischemic heart disease; DASH, Dietary Approaches to Stop Hypertension; AHEI, Alternative Healthy Eating Index; MDS, Mediterranean Diet Score; TJDS, Traditional Japanese Diet Score; PHDI, Planetary Health Diet Index; QUANTIDD, Quantitative Index for Dietary Diversity.

For MDS, β was −0.034 (SE = 0.026, *p* = 0.195), and the 1-SD MRR was 0.955 (95% CI, 0.892–1.023). For TJDS, β was −0.022 (SE = 0.013, *p* = 0.098), and the 1-SD MRR was 0.942 (95% CI, 0.878–1.011). For PHDI, β was −0.004 (SE = 0.004, *p* = 0.303), and the 1-SD MRR was 0.963 (95% CI, 0.897–1.034). For QUANTIDD, β was −0.483 (SE = 0.657, *p* = 0.464), and the 1-SD MRR was 0.965 (95% CI, 0.878–1.061) (Table 2, Figure 2a).

In the comparison of model fit, the DASH model had the lowest AIC and highest Akaike weight (0.434), followed by the AHEI model (0.341; ΔAIC = 0.482). The marginal R^2^ was slightly higher for the DASH model, with a difference of 0.001 between the two models (Table 3). These results indicate somewhat greater AIC-based support for the DASH model than for the AHEI model, whereas the difference in marginal R^2^ between the two models was small. In pairwise comparisons of standardized effect estimates, no statistically significant differences were observed between DASH and AHEI or between either score and the other dietary scores after Holm adjustment. The ratio of the 1-SD MRRs for DASH versus AHEI was 1.006 (95% CI, 0.947–1.070; Holm-adjusted *p* = 1.000) (Supplementary Table S11).

Significant dietary score × year interactions were observed for AHEI, TJDS, and QUANTIDD, but not for DASH, MDS, or PHDI (Supplementary Table S12). The interaction coefficients were negative for AHEI and QUANTIDD and positive for TJDS, indicating temporal variation in the associations between these baseline dietary scores and IHD mortality over the study period.

Sensitivity analyses generally supported the robustness of the inverse associations observed for DASH and AHEI, but the estimates for some of the other dietary scores were more sensitive to adjustment for obesity prevalence (Supplementary Table S10). Excluding 2010 mortality, additionally controlling for alcohol consumption or the proportion of the Muslim population, and excluding the extreme QUANTIDD value produced estimates broadly similar to those of the primary analysis. When obesity prevalence was omitted from the covariate set, the inverse associations for DASH and AHEI remained, with MDS, TJDS, and PHDI also showing significant inverse associations; the association for QUANTIDD remained non-significant.

In the supplementary all-cause mortality models, for TJDS, β was −0.020 (SE = 0.007, *p* = 0.003), and the 1-SD MRR was 0.948 (95% CI, 0.916–0.981). For QUANTIDD, β was −0.748 (SE = 0.321, *p* = 0.021), and the 1-SD MRR was 0.946 (95% CI, 0.904–0.991). For DASH, β was −0.003 (SE = 0.005, *p* = 0.491), and the 1-SD MRR was 0.987 (95% CI, 0.951–1.024). For AHEI, β was 0.000 (SE = 0.002, *p* = 0.847), and the 1-SD MRR was 0.996 (95% CI, 0.956–1.038). For MDS, β was −0.024 (SE = 0.013, *p* = 0.065), and the 1-SD MRR was 0.968 (95% CI, 0.936–1.002). For PHDI, β was −0.001 (SE = 0.002, *p* = 0.661), and the 1-SD MRR was 0.992 (95% CI, 0.958–1.028). The 1-SD MRRs were lowest for TJDS and QUANTIDD, whereas the estimates for DASH, AHEI, MDS, and PHDI were closer to 1.0 (Table 2, Figure 2b). These contrasting findings suggest that the dietary characteristics captured by different dietary scores have different associations with mortality outcomes.

## 4. Discussion

In this cross-country study with repeated annual mortality observations from 2010 to 2021, we compared six baseline food supply-based dietary scores calculated from 2010 food supply data in relation to IHD mortality within a common analytical framework. Higher baseline DASH and AHEI scores were associated with lower IHD mortality, with 1-SD MRRs of 0.914 and 0.908, respectively. Although DASH and AHEI showed relatively favorable model-fit indices, their standardized effect sizes showed no statistically significant differences when compared to those of the other dietary scores. In the supplementary analysis of all-cause mortality, TJDS and QUANTIDD were associated with lower mortality, showing a different pattern of associations from that observed for IHD mortality. The inverse associations for DASH and AHEI were generally robust across the sensitivity analyses. When obesity prevalence was omitted from the model, the estimates for MDS, TJDS, and PHDI became more inverse and their 95% CIs no longer included 1.0, suggesting that adjustment for obesity may attenuate the estimated associations for some dietary scores and that possible over-adjustment cannot be excluded. Further, significant score × year interactions for AHEI, TJDS, and QUANTIDD indicated that some associations with IHD mortality varied over the years. Because dietary scores were calculated from 2010 food supply data and treated as fixed baseline exposures, these interactions should be interpreted as temporal variation in associations with baseline dietary patterns rather than effects of changes in diet over time. As this was an ecological study, these country-level associations should not be interpreted as individual-level causal effects, and residual confounding cannot be ruled out.

The findings for DASH and AHEI in the present study were directionally consistent with those of individual-level cohort studies and meta-analyses showing associations between these indices and cardiovascular disease [13,25,28,42]. Both scores share the characteristic of assigning higher scores to dietary patterns with greater amounts of fruits, vegetables, grains, legumes, and nuts and lower amounts of sugar, meat, and sodium. DASH was developed to lower blood pressure [26,43], whereas AHEI was designed to comprehensively evaluate dietary factors associated with chronic disease risk [44]. These shared cardiometabolic features may partly explain the inverse associations of DASH and AHEI with IHD mortality observed in this study. Because we did not conduct analyses excluding individual components or component-specific analyses, we could not determine whether particular foods or nutrients, including sodium, contributed more strongly to the observed associations than the overall food supply pattern. Moreover, although DASH and AHEI showed inverse associations with IHD mortality, their standardized effect sizes did not significantly differ from those of the other dietary scores. Therefore, the present findings indicate country-level associations between stronger DASH- or AHEI-type food supply profiles at baseline and lower IHD mortality during 2010–2021, rather than individual-level effects or statistical superiority of these scores.

The estimates for baseline MDS, TJDS, PHDI, and QUANTIDD also indicated inverse associations with IHD mortality, although their 95% CIs included 1.0 in the primary analysis. Direct pairwise comparisons showed that the standardized effect sizes of these scores did not differ significantly from that of DASH or AHEI. However, the absence of significant associations for these scores should not be interpreted as evidence that their associations with IHD mortality were weaker. Rather, differences in the constructs and contexts represented by the individual scores may be relevant when interpreting the observed country-level estimates and their uncertainty. MDS and TJDS were originally developed to characterize dietary patterns within specific cultural contexts, and the same score may represent different combinations of foods when applied across countries with diverse food cultures. In addition, several components, such as olive oil in the MDS and rice and soybeans in the TJDS, vary substantially across geographic regions and may be correlated with socioeconomic, environmental, and other regional characteristics. Accordingly, the present findings should not be interpreted as contradicting previous evidence supporting the cardiovascular benefits of Mediterranean and Japanese dietary patterns [45,46,47]. PHDI and QUANTIDD, in contrast, capture somewhat different dietary constructs. The PHDI reflects a broader framework that integrates environmental sustainability and human health [33,34], and even when based on the same EAT–Lancet reference diet, the composition of a score and its associations with health outcomes may differ according to how the diet is operationalized and scored [48]. QUANTIDD primarily reflects the breadth and evenness of food-group distribution. Although greater dietary diversity assessed using QUANTIDD has been associated with longer healthy life expectancy in international comparative research [37], diversity scores do not necessarily distinguish cardioprotective foods from foods that are less favorable for cardiometabolic health [49]. Therefore, these differences in cultural applicability and the assessed constructs should be considered when interpreting the associations of these dietary scores with IHD mortality.

Baseline DASH and AHEI scores were associated with lower IHD mortality, whereas their corresponding estimates for all-cause mortality were closer to the null. In contrast, TJDS and QUANTIDD were associated with lower all-cause mortality in the supplementary analysis. Thus, different patterns of association were observed across the two mortality outcomes. However, differences in statistical significance alone cannot establish whether the associations differ between outcomes or are specific to IHD. Therefore, these findings should be interpreted as descriptive, hypothesis-generating country-level patterns rather than evidence of outcome-specific effects of particular dietary scores.

Some methodological and interpretative limitations should be considered when interpreting the findings of this study. First, this was an ecological study using country-level data. Consequently, country-level associations between food supply and IHD mortality should not be directly interpreted as associations between individual dietary intake and IHD mortality risk, as doing so may result in an ecological fallacy.

Second, although multiple country-level covariates were included in the models, residual confounding cannot be excluded. We considered the GBD Healthcare Access and Quality Index as a measure of healthcare access; however, a value corresponding to the 2010 baseline was not available in the dataset, and therefore healthcare access could not be included as a baseline covariate. In addition, we were unable to account for medication use, the diagnosis and treatment of dyslipidemia and hypertension, or within-country socioeconomic inequalities.

Third, because dietary scores originally intended to be calculated from individual-level dietary intake were applied to country-level Food Balance Sheet data, the validity of the exposure assessment may be limited. Food supply is not equivalent to actual individual intake and does not adequately reflect losses and waste at the retail and household levels, within-household allocation, or changes in nutrient composition associated with cooking and processing. In addition, for some score components, proxy operationalization using the available food groups was unavoidable. In global comparisons, staple foods and the degree of grain refinement vary by country, making it difficult to evaluate whole grains using consistent definitions. Therefore, we prioritized international comparability by scoring total grain supply without distinguishing whole grains from refined grains. However, this operationalization may not adequately capture the qualitative aspects of whole grains or dietary fiber. Likewise, because low-fat milk alone could not be extracted from the FAOSTAT Food Balance Sheets, we used “milk,” representing dairy products overall, for scoring. This operationalization does not fully reproduce the original score component based on fat content and may have led to misclassification of dietary scores. We also used sugars and sweeteners as proxies for sugar-sweetened beverages and olive oil as a proxy for the fatty acid ratio. Therefore, these proxy operationalizations meant that the scores used in this study did not fully reproduce the original scores and may have introduced deviations from the intended constructs and exposure misclassification.

Fourth, dietary scores were calculated from 2010 food supply data and treated as time-invariant baseline exposures throughout the 2010–2021 study period. Therefore, temporal changes in food supply composition or diet quality after baseline were not captured. Accordingly, the present findings should be interpreted as associations between baseline national food supply patterns and IHD mortality over the study period, rather than associations between changes in diet quality and changes in IHD mortality. In countries where food supply structures changed substantially after 2010, the baseline scores may have become less representative of subsequent food supply patterns over time, potentially attenuating the observed associations with IHD mortality or biasing them in other directions.

Fifth, the GBD IHD and all-cause mortality rates used as outcomes in this study were not based solely on directly observed data from each country; rather, they were estimates derived from multiple data sources, including vital registration and surveys, together with statistical models. Therefore, they entail uncertainty arising from differences in the availability and quality of mortality registration data across countries and from the modeling process. Such measurement error and uncertainty may attenuate the associations; however, if their magnitude or direction is related to food supply or national socioeconomic characteristics, they may have affected the direction and magnitude of the observed associations in unpredictable ways.

Finally, potential heterogeneity in the associations between dietary scores and IHD mortality by geographic region or income level was not specifically examined and warrants further investigation.

The findings of this study do not imply that DASH or AHEI should replace culturally appropriate dietary guidelines in all countries. Rather, when country-level food supply data are used for international comparisons of IHD, scores emphasizing established cardiometabolic dietary targets, such as DASH and AHEI, may be particularly relevant for characterizing country-level associations with IHD mortality. However, the direct comparisons in this study did not establish their statistical superiority over broader diversity or culturally specific dietary pattern scores. Future studies should focus on repeated measurements and temporal changes in dietary scores, analyses stratified by region and income, component-specific analyses, and associations with intermediate risk factors, such as blood pressure, low-density lipoprotein cholesterol, diabetes, and obesity. It is also important to link FAOSTAT supply data with individual-level dietary surveys to validate country-level indicators. For indices with multiple possible operationalizations, the robustness of findings across alternative scoring methods should also be assessed.

## 5. Conclusions

Among several country-level food supply-based dietary scores, higher baseline DASH and AHEI scores were associated with lower IHD mortality during the 2010–2021 study period. Their standardized MRRs and model-fit indices indicated similar magnitudes of association and broadly comparable model performance; however, their standardized effect sizes did not significantly differ from those of other scores. In the supplementary analysis, a different pattern of associations was observed for all-cause mortality, suggesting that dietary scores developed for different purposes may show varied associations depending on the health outcome and analytical context. Therefore, the target health outcome, cultural applicability across populations, and type of dietary data available should be considered when selecting and interpreting dietary scores.

## Figures and Tables

**Table 1:** Characteristics of the 148 study countries in 2010.

	Mean	SD	Percentile				
			5th	25th	50th	75th	95th
IHD mortality (/100,000)	145.26	90.59	49.90	84.13	118.67	175.80	343.46
All-cause mortality (/100,000)	932.97	444.35	438.27	627.40	844.01	1168.10	1662.73
DASH	23.92	3.83	18	22	24	27	30
AHEI	53.38	8.85	40	48	53	61	67
MDS	3.96	1.34	2	3	4	5	6
TJDS	−0.14	2.68	−4	−2	0	1.25	5
PHDI	87.24	9.26	73.2	81	85.5	94	103.5
QUANTIDD	0.87	0.07	0.74	0.84	0.89	0.93	0.95
Population (million)	46.37	152.83	1.65	4.85	10.99	32.46	145.30
Aging rate (%)	7.97	5.46	2.48	3.54	5.56	12.40	17.65
GDP (1000 US$/capita)	15.51	15.62	1.11	3.24	9.67	21.52	45.67
Years of education (years)	8.03	3.29	2.62	5.12	8.33	10.77	12.79
Smoking rate (%)	16.79	9.19	4.43	8.11	16.05	23.59	32.53
Physical activity (1000 METs-min/week)	2.68	0.79	1.55	2.04	2.69	3.15	3.96
Obesity rate (%)	15.33	8.96	2.75	6.84	15.61	22.05	29.36
Total energy supply (1000 kcal/day)	2.81	0.47	2.13	2.47	2.83	3.18	3.57
Sodium intake (g/day)	3.29	0.78	2.59	2.67	3.14	3.52	5.11
Muslim population (%)	27.23	38.05	0.00	0.00	4.60	56.80	98.79
PM2.5 (µg/m^3^)	27.52	14.85	9.32	18.50	23.09	33.88	55.58

Abbreviations: SD, standard deviation; IHD, ischemic heart disease; DASH, Dietary Approaches to Stop Hypertension; AHEI, Alternative Healthy Eating Index; MDS, Mediterranean Diet Score; TJDS, Traditional Japanese Diet Score; PHDI, Planetary Health Diet Index; QUANTIDD, Quantitative Index for Dietary Diversity; GDP, gross domestic product; PM2.5, fine particulate matter. Dietary scores: Possible scores range from 8 to 40 for DASH, 0 to 90 for AHEI, 0 to 8 for MDS, −8 to 8 for TJDS, 0 to 140 for PHDI, and 0 to 1 for QUANTIDD; higher scores indicate greater adherence to the corresponding dietary pattern.

**Table 2:** Associations of dietary scores in 2010 with IHD and all-cause mortality from 2010 to 2021 using covariate-adjusted linear mixed-effects models.

	β	SE	*p* Value	MRR Per 1-SD Increase	95% CI Lower	95% CI Upper
IHD mortality						
DASH	−0.024	0.010	0.016	0.914	0.849	0.983
AHEI	−0.011	0.005	0.021	0.908	0.838	0.985
MDS	−0.034	0.026	0.195	0.955	0.892	1.023
TJDS	−0.022	0.013	0.098	0.942	0.878	1.011
PHDI	−0.004	0.004	0.303	0.963	0.897	1.034
QUANTIDD	−0.483	0.657	0.464	0.965	0.878	1.061
All-cause mortality						
DASH	−0.003	0.005	0.491	0.987	0.951	1.024
AHEI	0.000	0.002	0.847	0.996	0.956	1.038
MDS	−0.024	0.013	0.065	0.968	0.936	1.002
TJDS	−0.020	0.007	0.003	0.948	0.916	0.981
PHDI	−0.001	0.002	0.661	0.992	0.958	1.028
QUANTIDD	−0.748	0.321	0.021	0.946	0.904	0.991

Only the main effects of dietary scores are presented; coefficients for the covariates are not shown. β represents the regression coefficient for each dietary score in the model of the log-transformed age-standardized rate. Baseline dietary scores and continuous country-level covariates were mean-centered by subtracting their respective means across the included countries. Models were controlled for year, GDP, aging rate, total energy supply, years of education, smoking rate, physical activity, PM2.5, obesity rate, and latitude. **Abbreviations:** MRR, mortality rate ratio; CI, confidence interval; SE, standard error; IHD, ischemic heart disease; DASH, Dietary Approaches to Stop Hypertension; AHEI, Alternative Healthy Eating Index; MDS, Mediterranean Diet Score; TJDS, Traditional Japanese Diet Score; PHDI, Planetary Health Diet Index; QUANTIDD, Quantitative Index for Dietary Diversity.

**Table 3:** Model fit statistics for linear mixed-effects models including each dietary score.

	DASH	AHEI	MDS	TJDS	PHDI	QUANTIDD
AIC	−5764.297	−5763.815	−5760.220	−5761.244	−5759.588	−5759.071
ΔAIC	0.000	0.482	4.077	3.053	4.709	5.226
Akaike weight	0.434	0.341	0.057	0.094	0.041	0.032
BIC	−5676.583	−5676.101	−5672.506	−5673.530	−5671.874	−5671.357
Marginal R^2^	0.576	0.575	0.564	0.564	0.562	0.560
RMSE	0.027	0.027	0.027	0.027	0.027	0.027

**Abbreviations:** DASH, Dietary Approaches to Stop Hypertension; AHEI, Alternative Healthy Eating Index; MDS, Mediterranean Diet Score; TJDS, Traditional Japanese Diet Score; PHDI, Planetary Health Diet Index; QUANTIDD, Quantitative Index for Dietary Diversity; AIC, Akaike information criterion; BIC, Bayesian information criterion; RMSE, root mean square error. ΔAIC represents the difference between the AIC for each model and the lowest AIC among the candidate models. Akaike weights indicate the relative support for each model among the candidate models. Marginal R^2^ represents the proportion of variance explained by the fixed effects. RMSE values are presented to three decimal places; small differences among models were observed only at higher decimal precision.

## Data Availability

The data used in this study were obtained from publicly accessible databases, including the Global Burden of Disease (GBD) Study, FAOSTAT, and the World Bank. The source data are available from the respective data providers and repositories, as described in the Methods section and cited references. The datasets analyzed in this study were derived from these publicly available sources.

## Supplementary Materials

The following supporting information can be downloaded at https://www.mdpi.com/article/10.3390/nu18203456/s1, Table S1: Overview of dietary indices and scoring methods used in this study; Table S2: Components and scoring criteria of the modified DASH score used in this study; Table S3: Components and scoring criteria of the modified Alternative Healthy Eating Index used in this study; Table S4: Components and scoring criteria of the modified Mediterranean Diet Score used in this study; Table S5: Components and scoring criteria of the Traditional Japanese Diet Score used in this study; Table S6: Components and scoring criteria of the Planetary Health Diet Index used in this study; Table S7: Food groups and calculation of the Quantitative Index for Dietary Diversity used in this study; Table S8: Country-specific dietary scores in 2010; Table S9: Country-specific ranking of ischemic heart disease mortality rates in 2010. Table S10: Sensitivity analyses of the associations between baseline dietary scores and ischemic heart disease mortality; Table S11: Pairwise comparisons of standardized effect estimates for dietary scores and ischemic heart disease mortality; Table S12: Interaction between baseline dietary scores and year in relation to ischemic heart disease mortality.

## Abbreviations

The following abbreviations are used in this manuscript:

IHD	Ischemic Heart Disease
DASH	Dietary Approaches to Stop Hypertension
AHEI	Alternative Healthy Eating Index
MDS	Mediterranean Diet Score
TJDS	Traditional Japanese Diet Score
PHDI	Planetary Health Diet Index
QUANTIDD	Quantitative Index for Dietary Diversity
MRR	Mortality Rate Ratio
AIC	Akaike Information Criterion
GBD	Global Burden of Disease Study
SD	Standard Deviation
LMM	Linear Mixed Models
1-SD MRR	Mortality Rate Ratio per 1-SD
CI	Confidence Interval
